# Don Quixote: A Psychological Study

**Published:** 1859-01-01

**Authors:** J. M. Guardia


### Art. VIII.—DON QUIXOTE: A PSYCHOLOGICAL STUDY.

BY DR. MOREJON.

From the French of Dr. J. M. Guardia.

"As there are an infinity of wise things which are done in a very foolish manner, so there are foolish things which are done in a very wise manner."—Montesquieu.

A volume might be written on the instances of madness transmitted to us by antiquity. I do not speak now of the extravagances and absurdities abounding in the biography of conquerors, emperors, and kings, which belong to the department of history and ethics, but solely of the aberrations of intelligence, which enter into the domain of mental medicine. Nothing is better known, for example, than the mania of Thrasyllus of Æxone (a district of Attica), who thought himself proprietor of all the ships of the Piræus; and everybody has read in Horace the amusing history of that inhabitant of Argos, who seated alone at the theatre, applauded, with all his strength, an imaginary play and absent actors. A king of Persia, Xerxes, was enamoured of a plane tree. A young man of Athens fell in love with a statue of Good Fortune, and died in consequence. The physician Menecrates pretended to be Jupiter, and Philip, king of Macedon, could not cure him of his conceit. Ælian, to whom we owe many of these instances, speaks also of a fool who amused himself with counting the waves of the sea, and of an idiot who sought a staircase in a pitcher (*qui cherchait un escalier dans une cruche*).*

These isolated facts are curious, but less interesting than those instances of epidemic madness, so frequent in ancient times. I might cite many, but will only mention the gaiety of the Tirynthians, reported by Athenæus (Book VI.), after Theophrastus, and the delirium that Lucian † has described, with much wit and malice.

* Ælian: *Hist. var.* xiii. 17.

† Lucian: *Quomodo hist. sit conscrib.*, 1.

He recounts that at the end of a representation of the Andromeda of Euripides, given to the inhabitants of Abdera in broad daylight, in the middle of summer, all the spectators were seized with an attack of burning fever. When the fever was characterized by profuse perspiration, and bleeding from the nose, the sick began to run in the streets, declaiming with a loud voice the *rôle* of Perseus, in imitation of Archelaus, a famous actor of the time, who had played in the piece. Lucian gives an ingenious, and almost medical explanation of this singular epidemic (πανδημεὶ ἅπαντας), of the manner of its attack, of the transformation it underwent, and of the crisis which terminated it. At the beginning of winter a sharp frost, which came on suddenly, put an end to the delirium of the Abderites. Democritus was well avenged of the folly of his fellow-countrymen.

It is known that this illustrious philosopher, given up to the contemplation of nature, passed his life in solitude, a stranger to the things of the world, or only mixing with them to show the absurdity of them. This wisdom had the appearance of madness. People were afraid of it, and sent for Hippocrates. The doctor arrived, conversed with the invalid, and soon perceived that it was not necessary to administer hellebore. He had rightly guessed. Bordeu remarks, acutely, that in this case, it was medicine which judged philosophy, and that philosophers would do wrong to forget it. He pretends also that philosophers have never been able to impose on physicians, who go straight to the causes of things.*

However this may be, the author of the *Letter to Damagetus* (an apocryphal article in the Hippocratic collection), where this history is recounted at length, pretends that Hippocrates found Democritus occupied in seeking in the entrails of animals, the nature and causes of madness, and the means of curing it. But he would have had considerable difficulty in arriving at a conclusion, if it is true that he considered this world to be a large lunatic asylum, of which the inhabitants pass their time in mocking at one another, while each is ignorant of his own condition. This was precisely the subject of his witticism. It will be seen that Democritus resembles certain philosophers of our time, who, standing very high in their own estimation, consider the rest of mankind as actors in a pleasant comedy, and amuse themselves with this spectacle as a relaxation from the greatness of their own thoughts. He differs from them altogether in one respect—that he was charitably willing to correct those errors of humanity, which serve as diversion to these brilliant wits. In his opinion, man from his birth till his death was incessantly diseased. It has been remarked on this subject, that he also must have been in the same case; so

* Bordeu: *Rech. sur les maladies chron.*, t. II, p. 805, des Œuvres complètes.

that however we may admire his wisdom, we may apply to him the words of La Fontaine—

"Il connait l'univers et ne se connait pas."

Democritus places the seat of madness in the liver, and its cause in the bile. Aristotle has remarked after him, that the greater number of men who have distinguished themselves in arts, in letters, or in science, have been of a melancholy temperament. Atrabiliousness played a prominent part in the ancient theories concerning mental aberration, and the word melancholy still belongs to science. This observation is singular; it will serve to explain the ancient saying, "There is no man so great but he has his grain of madness."

A fortune is often in a word, and so it ought to be: there are many small wits who seek to console themselves with their mediocrity. Unhappily, men of imagination and intelligence are come to their aid, and impelled by very different motives, they have endeavoured to include in the annals of mental malady, famous sages and illustrious *savans*, such as Socrates and Pascal.

This is not the place to examine the expediency and the utility of those retrospective researches, the fruit of an erudition more ingenious than patient, to which a solid base is always wanting,—that of personal experience and clinical observation. To endeavour to demonstrate the madness of those great minds who have astonished the world by the example of their life, or by the sublimity of their works, would be an ungrateful task, if it were not a dangerous paradox. Besides, there is in the examination of these delicate questions, an essential point which must not be neglected. It is necessary to inquire attentively into the general ideas, the beliefs, the opinions, the mental condition of the contemporaries of these great men, for the greatest men are before their time, and the opinion of the crowd exercises a permanent and positive influence upon them. Since this is the case, it is more important, perhaps, to pay attention to the mass than to individual instances, the observation of whom belongs, but indirectly, to the general history of epidemic maladies.

Precisely thus has Cervantes proceeded. He attempted to cure his country of a chronic malady, which presented all the characters of an epidemic. The reading of the romances of chivalry, had long perverted the taste of the public, and turned their heads. The great discoveries, the distant expeditions, and the prodigious conquests of the Spaniards, in the fifteenth and sixteenth centuries, contributed not a little to favour the spirit of adventure, and of heroic extravagance, which finally resulted in the decline of the Spanish monarchy. Some philosophic commentators have suggested—not without apparent reason—that the Chevalier

Don Quixote is none other than Spain herself, already distempered and dreaming, on the brink of ruin, of that universal empire, which she pursued so madly without being able to obtain it. This is not the place to decide whether the preceding hypothesis is well-founded, or otherwise. However the case may be, Cervantes has written the history of a lunatic, the celebrity of which is great in the republic of letters. For two and a-half centuries, it has delighted all who knew how to read it. Its renown has carried it to the four quarters of the world, and it may be said without fear of exaggeration, that after the Bible, Don Quixote is of all books the most universally known and appreciated. No ancient author has had a greater number of interpreters. However, the commentators have not said all that may be said, and the subject is so far from being exhausted, that the last comer finds always something to glean in this fertile field. Dr. Morejon (Don Antonio Hernandez) has enjoyed this good fortune. In his great posthumous work, *sur l'Histoire Bibliographique de la Médecine Espagnole*, this author, one of the most learned men of the age, has consecrated to the immortal work of Cervantes a new and special article, which illustrates in its most favourable aspect the ingenious history of the Chevalier of La Manche. He there demonstrates with great acuteness that medicine should claim Cervantes as rightly belonging to it; and for my part I think, after having read his plea, that the faculty may adopt it, and be proud of it. If Molière has exercised a beneficial influence upon the dignity of the medical profession by his keen satire upon charlatans and quacks, how much more does Cervantes deserve the gratitude of all true physicians—he who has never spoken evil of them, and who has done such signal service to science.

These considerations have led me to think that many who have devoted themselves to the study of mental maladies would be glad to read this curious commentary. I have therefore translated it from the Spanish original with all possible fidelity. I have only allowed myself to omit one page of introduction and encomium, which suppression will have the effect of abridging somewhat this memoir.

In the last section of his *Histoire de la Médecine Espagnole au XVI. siècle*,* Dr. Morejon expresses himself thus:—

"This history would lose its greatest ornament if I neglected to mention Cervantes. He well deserves to find a place there on account of the medical observations which adorn his greatest book, and which have almost escaped the attention of his most ardent admirers.

* *Historia bibliografica de la medicina española*, tomo II, parte 7, siglo XVI, § xx. CONCLUSION.—*Bellezas de medicina practica descubiertas en la obra de Cervantes*, pp. 166-180.

"The astonishing abilities of Cervantes, his fruitful imagination, the richness and grace of his style, the end which he proposed to himself, and which he attainedin writing his immortal *Don Quixote*, viz., the ruin of the chivalrous romances, a style of reading equally pernicious and frivolous—these are titles which have rendered his name glorious in the world. He deserves the praises of medical men also for the ability he has displayed in the description of the kind of madness known under the name of monomania" (no; the mania of Don Quixote was multiform).

"Many learned men, national and foreign, have devoted their talent and learning to the critical examination of the work of Cervantes. The analysis made by the Spanish Academy at the commencement of the magnificent edition of 1780, deserves to be read for all which concerns the subjects on which it treats, whether one would study in Cervantes, considered as a writer, the invention of the fable, the qualities of the action, the characters of the personages, the merit of the narration, the propriety of the style, or the utility of the moral. Still, to make this analysis complete, a profound knowledge of medical philosophy is necessary, a knowledge naturally foreign to this illustrious company. They may well place Cervantes in the same rank as Milton, Virgil, and Homer; but they could not appreciate nor make known his great merit in the description of that form of mental alienation of which he has treated, and in which he surpasses the celebrated Aretæus, the greatest painter of maladies, the Raphael of medicine; *y á quien por su habilidad en este ramo se la conoce por el Rafael de la medicina.*

"Moses and Homer have each been honoured by a dissertation; the first, thanks to some very limited notions of chemistry, the second, for his anatomical acquirements, although very imperfect. Thucydides, Virgil, and Suetonius, for having described the plague, are praised by physicians, and cited as models in the description of pestilential maladies. Montesquieu holds also his place in the history of medicine, in consequence of his theory of the influence of climates on institutions—a theory which he has copied from the Spaniard Huarte. And does not Cervantes still better deserve to be offered to the Spanish student as a model for the description of disorders of the mind? To examine this question, we must analyse the predisposition, the occasional causes, the development, the progress, the treatment, the prognosis, and the termination of the madness of the famous Don Quixote —a novel affection in the history of mental alienation, and created by the brilliant and fruitful imagination of the Spaniard Cervantes Saavedra.

"There is no hospital or asylum for lunatics where one may not see some insane person who thinks himself pope, king, cardinal, bishop, general, or captain, count, duke, or marquis—poor, or rich and powerful, possessed with a spirit good or bad. But we do not find in the annals of insanity a lunatic so benevolent, so amorous, so desirous of the public good, as the knight-errant, who wishes to banish from the world all evil-doers, fools, and cowards, with all the wrongs and iniquities that they have committed, and to pour a consoling balm upon the sufferings and labours of the unfortunate; in a word, the hero who wished to disenchant the unrivalled Dulcinea del Toboso, whose madness, described by the enchanting pen of Cervantes, in a manner so exact, and so true, has proved that he was right in saying that history written in this way enjoyed immortality—very different from that which, for want of these ornaments, soon passes from life to death. Let us analyse, then, the madness of Don Quixote under all its aspects, without losing sight of the conditions which permit the philosophical physician to form for himself a complete idea of a malady according to the laconic and profound precept of Hippocrates,—'that it is necessary in the study of diseases to take account of their qualities, causes, forms, seat, development, the time of their continuance, and their termination.'

"Cervantes had to describe a particular kind of madness. He begins by studying the circumstances (*condicion*) and the habits of his subject, the species, the character, and the nature of the affection that he is going to depict, including the predisposing and occasional causes which most contributed to its development. He marks its seat, reviews its periods, its changes, and its termination. He reasons upon the prognosis, adopts the most suitable mode of treatment, conforming himself so exactly to the rules of art, that he may serve as a model to all philosophical students of medicine.

"The details which form the *ensemble* of this medical history are arranged in such perfect proportion, and in such harmonious combination, that they result in a perfection of beauty equally charming and attractive.

"*Predisposition and Causes.* Conditions which predispose to madness: 1. Temperaments, bilious and melancholy.—Don Quixote was 'tall of stature, of a robust constitution; his visage thin, his skin shrivelled and hairy.' 2. Ripe or mature age.—Don Quixote 'touched on his fiftieth year.' 3. A penetrating and cultivated mind.—Don Quixote had wit, an excellent memory, and a good education. He possessed all the accomplishments of a knight-errant—theology, jurisprudence, medicine, botany, astronomy, mathematics, history, and others besides. 4. Pride of race and nobility.—Don Quixote was a gentleman (*hidalgo*) of La Mancha, descended directly through the male line (*por linea recta de varon*) of Gutierre Guijada, the conqueror of the sons of the Comte de Saint-Pol. 5. Violent exercises.—Don Quixote was a great hunter of hares. 6. The transition from a life of activity to one of idleness.—Don Quixote neglected almost entirely the exercise of the chase, and even the management of his own affairs. 7. The use of a highly-flavoured diet, mucilaginous and difficult of digestion.—Don Quixote 'ate generally for supper hashed meat, pulse on Fridays, giblets on Saturdays, and stuffed pigeons on Sundays.' 8. The seasons of summer and autumn.—Don Quixote had his greatest fits of madness the 28th July, the 17th August, and the 3rd October. 9. Amorous passions.—Don Quixote was much enamoured. 10. Excess of reading.—Don Quixote sold several acres of arable land to buy books of chivalry and of erotic poetry. 11. Prolonged night-studies.—Don Quixote 'read without ceasing day and night, so that by reason of reading, and for want of sleep—other things conducing to the same result—his brain was so disordered that he lost his reason.' We find specified in these last words, with as much precision and clearness as Hippocrates or Boerhaave could have used, the diseased organ, the seat, and the immediate cause of the malady.

" *Symptomatology.* The word madness is generic; it embraces different kinds, and even varieties; the symptoms also are always related to the diversity of the causes which produce them. When once Don Quixote had completely lost his reason, he imagined that all he had read in books of chivalry, and in amorous poetry, was real. From that time he dreamed of nothing but of quarrels, battles, defiances, wounds, declarations and proposals of love, pains and cares, and other impossible extravagances. He got it fixed so firmly in his head, that all these dreams of the imagination—the fruit of his readings—were true, that there was no history to him more certain. Then he conceived the design of making of himself a knight-errant, and going round the world in search of adventures. This is the specific character of this strange and singular madness; the whole of these circumstances constitute what is called in medicine *le syndrome symptomatologique* (the enumeration of symptoms without necessary relation to determinate maladies). Thus the forms and the symptoms of the affection of Don Quixote are constituted by a series of successive attacks of arrogance, of pride, of courage, of fury, of audacity, which manifested themselves, each in their turn, during the course of his malady in each of its periods. It appears always, that the exterior objects which fell under the notice of the invalid, far from producing in him regular sensations or images, occasioned serious disturbances in his judgment, reproducing themselves in his imagination in a manner conformable to the interior disposition of his deranged brain.

" *Times and periods of the malady.* All diseases, without exception, the longest as well as the shortest, have their periods. Cervantes has not endeavoured to dispense in this case with the rule imposed by Galen. The first appearances, the increase, the persistence and the decline of madness, are indicated in a masterly manner in his work by the adventures and escapades of Don Quixote.

" The madness makes its appearance in the summer, and announces itself in this way. The hero talks to himself in his apartment of things concerning chivalry—analogous to the occasional causes of his complaint. He fences, sword in hand, against walls, as if trying to conquer giants, felons, and robbers, over whom he desired to triumph, to redress all wrongs, and to demand satisfaction for all injuries and offences.

" Afterwards he began to prepare all sorts of arms, and conceived the project of going all over the world, exercising the profession of a knight-errant—a project which he executed by his escapade of the 28th of the month of July, one of the hottest days of the season, and in the night of which the first violent attack of his insanity manifested itself, followed speedily by his meeting with the half-naked boy tied to the trunk of an oak, and the merchant of Toledo.

" The increase of the malady is marked in the first place with the second expedition of the ingenious hidalgo, until his return to his home. In this second interval took place the battle with the windmills; the encounter betweent he hero of La Mancha and the Biscayen; the adventures of the unmerciful jockeys, of the inn mistaken for a castle, of the funeral procession, of the fuller's mill, and of the helmet of Membrin; the deliverance of the galley slaves, the penance in the retreat of the Sierra-Morena, the fight against the leather bottles of red wine, and the skirmishes with the members of the Holy Hermandad and the flagellants. In the account of this period of aggravation, Cervantes engages irresistibly the admiration of every philosophical physician. In this part of his book he has, to my mind, described that kind, or rather that variety of mania, of which Aretæus has said, at the close of the chapter devoted to it, 'There exists another kind of delirium in which the sufferers tear their limbs, piously believing that it is the will of the gods, and that they are pleased with this conduct.' The picture painted by the Spanish author of the insanity of Don Quixote, imitating *le Beaux Tenébreux*, surpasses the original of the physician of Cappadocia.

"It is here that Cervantes has collected all the features which mark the greatest intensity of this malady—namely, incredible power of enduring long-continued watchings, prolonged and frightful abstinence from food, insensibility to the action of cold, profound sighs, tears, fervent prayers, a marked inclination to tear his clothes or to deprive himself of them, to remain in his shirt, to cut capers and throw somersaults, heels over head — developing enormously the strength of nerves and muscles—mortification of his body in honour of the goddess of his amours, the unrivalled Dulcinea.

"In the retreat of the Sierra Morena, a particular well worthy of the attention of medical philosophers is the meeting of Cardenio. In general the insane live apart, shunning each other, despising and ridiculing each other, neither sympathizing nor consorting together, except in so far as their delirium is analogous; and even in this case they quarrel about a trifle, but are easily reconciled. This fact precisely has been noted by Cervantes with a masterly hand, in the episode of this gallant young man, driven mad by believing that Don Fernando had carried away his idol, Lucinda. We see also an example of those lucid intervals continually presented by the insane. The account of his misfortunes given by Cardenio to the curate, in one of these intervals, deserves to be read in confirmation of this truth. Another trait which merits the attention of medical men is the custom which the insane have of changing their name. In the course of this period, Don Quixote takes the name of the Knight of the Rueful Countenance, and in the subsequent period that of the Chevalier of Lions.

"The shades which distinguish the alternatives of moral character in monomania are, pride, arrogance, an exaggerated sentiment of personal courage, and confidence in personal vigour. Don Quixote boasted on all occasions of the strength of his indefatigable arm; and on one occasion he went so far as to say to his esquire, that heaven had never created, and hell had never seen, any one who could intimidate him, or cause him to feel afraid.

"The last journey of the hero, until his defeat at Barcelona by the Knight of the White Moon, at the end of which he returned home for the third time, constitutes the period of persistence and decline of his madness. The symptoms of this period were: the cart of the parliament of Death, the fight with the Knight of the Looking-glass, the meeting with the lions, the cavern of Montesinos, the famous adventure of the enchanted bark, that of the afflicted duenna, the unequal fight with Tosilos, the battle with the bulls, the adventure of the beautiful Morisca, that of the pigs, of the enchanted head, and at last, that of the Knight of the White Moon, when the transition from one malady to another commences—a transition which the Greeks call *metaptosis*, and which is one of the most curious and difficult subjects of practical medicine.

"*Transformation of Madness.*—Diseases pass sometimes, or extend themselves from one organ to another, without any diminution of the primitive lesion; or they change from one place to another, the organ first injured remaining without damage, but always preserving the first essence of the evil. At other times they change both their seat and their nature, when a malady supervenes which differs from the first, —a question important to practical medicine, and unhappily little studied. Cervantes offers us an example of this transformation of disease. An acute fever attacked Don Quixote, and immediately all the physical and general characters of the primitive affection were altered. A curious alteration in three points of view: first, for practical medicine; then in respect to legal medicine, for without this transformation Don Quixote could not have made a will, or at least his testament would have been nul; lastly, by reason of the influence this change had on the prognosis and the termination of his disease.

"*Prognosis.*—The sudden change from madness to a bitter dejection, a profound melancholy, and the complication of an acute fever, the abrupt passage from madness to reason,—so many circumstances which must inspire fears for the life of the patient. These were, precisely and collectively, the phenomena which presaged the death of the celebrated knight.

"*Curative Plan and Moral Treatment.*—The greatest title of Pinel to glory is, on the testimony of Broussais, the application of moral treatment to mental derangement. But this glory is due to the Spaniards rather than to Pinel. This French author, in his valuable work, praises the method adopted in the lunatic asylum of Saragossa, where, before his time, this theory was put in practice. This idea Saragossa borrowed probably from Valence; and two hundred years before Pinel, Cervantes handled it magisterially with so much genius and ability, that one cannot but admire the medico-moral strategy which he employs to calm the fury and the transports of his knight-errant, —a means not less original than that which he used to banish from Spain the epidemic of bad taste which led everybody to read books of chivalry.

"To direct the moral treatment of melancholy and mania, it is necessary to study profoundly the human heart and intellect in general, and in particular that of the patient. Cervantes fulfilled these two conditions. He knew Don Quixote as well as if he had been his own son, and no one better than he could discover the means of relieving him.

"Six persons figure in the history, taking part in the treatment, with different rôles, to fill the two extremes of the epigraph of Boerhaave: the curate (a learned man), Master Nicholas, and Sanson Carrasco, favour the whim of the knight; the canon of Toledo, the governess, and the niece, dispute it vigorously.

"To commence the treatment, they decided first on the removal of the efficient cause of the disease; and in order to do this, they examined and burned the books of chivalry and love in a room, of which they blocked up the door, and they pretended that all this was done by enchantment: this was the most sensible conduct that could be adopted in such circumstances. The learned magician, Mugnaton, came in a cloud, riding on a serpent; he flew out of the roof, and left the house full of smoke.

"Such is the general precept applicable to all maladies; it may be said that it is a miracle to see one single person cured as long as the causes producing the disease continued to exercise their influence.

"However, they did not succeed at once in producing the desired effect; first, on account of the economy of the romance, of which the action terminated coldly at the cessation of the disease; and in the second place—and this is an observation important to our point of observation—in consequence of a trifling inadvertence of the niece, who confounded the name of Freston or Friton with that of Mugnaton; for it is necessary to proceed with much prudence and sagacity, the least negligence rendering all such plans abortive.

"It was by a ruse of this kind that the curate of his village, and the barber, found means to entice him from the Sierra Morena, where his extravagance reached its highest point. They disguised themselves at the inn; the curate with a doublet of velvet bordered with white satin, and the barber with a long beard, half-white half-red (the tail of a red ox); a disguise changed soon for another of the same sort, which they thought more effectual.

"The beautiful and unfortunate Dorothea throws herself at the feet of the knight-errant, and relates her troubles to him. She pretends to be the Princess Micomicone, and obtains from him a promise of satisfaction for her wrongs. By this excellent pretext they succeed in drawing the madman from the Sierra; they bring him to the inn, where he falls into a profound sleep, complicated with a kind of somnambulism known in Spain, analogous to the state of his exalted imagination, and prelude to a remission of his madness, during which they can, without much resistance, transport to his home the poor madman, in a cart drawn by oxen.

"The determination taken by the curate and the barber, to pass nearly a month without seeing the invalid, for fear of renewing the recollection of passed things, until he should begin to give evidence of the recovery of his reason,—this determination was excellent; and if he had remained without seeing any of his family or his own house, it would have been better still. The dietary regimen which was prescribed and followed was the most suitable.

"The invectives of the governess at the time when the madness begins to reappear; the threats she holds out to go and complain loudly to God and the king if he did not remain quietly at home, and keep good order there; and those of the niece, when she made him observe that all that he detailed about knights-errant was fable and falsehood, and that his histories, worthy of the fire, deserved at least the censure of the inquisition, or some mark which should brand them with infamy as destroyers of good morals,—these complaints and these menaces were very suitable means, and the most powerful in Spain, and thus they were used by the canon of Toledo.

"The third trick of this kind was the agreement of the curate and the barber with the bachelor Sanson Carrasco, who, disguising himself in his turn, under the name of the Knight of the Looking-glass, fought a first time with Don Quixote—not, however, with the success and satisfaction which he had in the second encounter at Barcelona, when he took the name of Knight of the White Moon.

"The same plan is followed until the approaching end of the complaint of Don Quixote, when he resolves to turn shepherd. The bachelor urges him strongly to bestir himself, and begin a pastoral life. He tells him that he has already composed an eclogue, and purchased from a farmer of Quintanar two famous dogs to guard his flock, answering to the names of Barcino and Butron.

"The last but one of the moral stratagems resulted in the weakening of the madness of Don Quixote—a weakening painted by Cervantes with so much exactitude and truthfulness, that he seems to have borrowed the pencil of the doctor of Cappadocia, if indeed the Spanish author has not improved upon his colouring: the terms are almost alike in both authors, but the last is the most brilliant in exposing the moral phenomena of the decline of madness.

"Not only did Cervantes precede Pinel in the moral treatment of madness, but Broussais himself, in the theory which has made him so many proselytes in Europe; for the Spanish author establishes the point that 'the stomach is the laboratory where health is manufactured,' and by these words concerning the madman of Seville, he shows that he knew the relations which exist between this organ and the perturbations of reason. But the man to whom he has for two centuries been giving a good lesson is Hahnemann—this modern sectary, who, under the ridiculous name of homœopathy, pretends in these days to dazzle inexperienced youth, giving as new a doctrine known for ages in Spain, where it has been used in a manner judicious and philosophic—very different from that adopted by this maker of systems.

"Cervantes himself avows that the only object of his book was to banish the objectionable love of reading books of chivalry, which were doing so much harm. This result, a doctor of La Mancha, Sanchez Valdes de la Plata, had not been able to accomplish; he had pursued the same design, in conformity with the general principles of medicine—*contraria contrariis curantur*. The author of 'Don Quixote,' penetrated with the truth of an observation of Hippocrates—viz., that diseases are often cured by similar causes to those which produced them—decided to make use of the method now called homœopathy.

"From the time of the Middle Ages to the Crusades, Spain was infected with the romances of chivalry. Cervantes composed also a chivalric romance, which was intended to make all the rest disappear, to cure the reason of its pernicious credulity, and to leave the immortal work to all classes of society, and especially to physicians, who may discover there still more beauties than I have been able to point out.

"Only one thing is wanting to my mind in the work of Cervantes to make the work complete—that is, a *post mortem* examination of Don Quixote. Perhaps he omitted to insert it because he was convinced of the insufficiency of pathological anatomy in this class of diseases, or because the lunatic, having recovered his reason, the dryness of the brain was no more the immediate cause, nor the seat of the cause transformed into another disease; therefore, in consequence, nothing would have been found corresponding to the wanderings of the imagination. Perhaps, also, the true motive was the impossibility of doing it, surrounded by the prejudices of his family and his neighbours—and especially in a village. We find no mark of this in the history of the Cid Hamet Benengeli. Notwithstanding this omission, the history of the ingenious hidalgo Don Quixote is described in accordance with all the rules of medicine; for there are not many physicians who, in the writing of diseases, remember all the scientific conditions necessary to the description of a morbid state—a thing arduous and difficult after Sydenham.

"There is in the work of Cervantes the same truth as in his imagination—order, clearness, careful imitation of nature, and an application of moral means, more ingenious and more appropriate to the cause of lunacy than any that would have been imagined by Pinel, or any of his predecessors. Until this time painting had not been applied to the service of medicine, except to represent the different stages of the *pellagra* (*rosa de Asturias*), of other diseases of the skin, and of some diseases of the eyes. Perhaps this idea is born in Spain, for I have seen at Madrid some very ancient pictures representing the different symptoms and stages of the *mal de St. Lazare* (*mal de Sar. Lazaro*, a kind of leprosy—this term is applied sometimes to *teigne*)—a disease so widely spread formerly amongst us that there were many hospitals for its special treatment; but it is happily now almost extinct.

"In its turn, the skill of the engraver is employed to preserve to us the features of chivalric extravagance displayed in the malady of Don Quixote. Some of these characteristics strike us with admiration and surprise. A single man attacking two imaginary armies; the adventure with the fuller's mill, of which the terrible crash in the middle of a dark night would have struck terror into any other heart than that of Don Quixote; the descent to the cavern of Montesiros, which surpassed the descent of Eneas into Hell (the author adds, *in search of Creusa, his wife*), and which the historian, Cervantes, describes with as much art and sublimity as the Poet of Mantua, giving us in the same way an example of that asphyxia so common among divers and those who descend into deep places.

"Let medical men, then, study 'Don Quixote,' not only for a moment's amusement after the labours of the day, but for the sake of contemplating the work of a genius in the description of menta diseases. To observe how completely Cervantes had present to his mind all the conditions requisite to this kind of investigation, and to consider with what ability he has described a new kind of madness, and how he has succeeded in making this lunatic interesting without making him ridiculous, so that his hero, among all his extravagances, inspires a secret interest in the success of his chivalric adventures.

"Let them examine in this history the lucid intervals or periods of calm, and they will there find all the proper characteristics—increase of memory, *bon-mots*, and sallies of wit—that is to say, the moral features which distinguish the malady with the remains of a good education, the politeness and urbanity of the hidalgo. Thus he is seen in the palace of the Duke, and at the house of Don Antonio Moreno, at Barcelona, altogether transformed, with all the distinction and courtesy of a chevalier; and even in his conversations, in his stories, and in the episodes which embellished the work, giving lessons and precepts to all classes of society. A new tribute of admiration to be paid by the medical profession in addition to all those merited by this great genius!

"Immortal shade of Cervantes! In the midst of the profanities which doctors dare to utter—in the midst of so many detractors among the members of the most benevolent of professions—thou livest for them—thou dost distinguish with consideration and respect the men of learning, wisdom, and talent, regarding them as divine. Receive, then, the tribute of gratitude; and while art and literature elevate thee to the most envied pinnacle of glory, I will consecrate a more durable monument to thine honour in giving thee a place in the history of Spanish medicine!"

Montesquieu has remarked, in speaking of the Spanish, "The only good book they have is that which shows the absurdity of all the rest." It was scarcely possible to give Cervantes better praise at the expense of the most illustrious of his predecessors and successors. But while this may be true, it must be conceded that a nation may well console itself, if it possesses but one book that is worth a whole library. The testimony of Montesquieu, who can never be accused of partiality to Spain, deserves, however, some consideration. We might oppose to it, if necessary, the feeble arguments of some commentators who have thought, I know not why, that Cervantes painted himself in the portrait he sketched of Don Quixote—an hypothesis by no means probable, if we bear in mind that by the side of this insane hero the author has taken care to represent, in the person of the esquire, Sancho Panza, good, popular, or common sense. This consideration alone is sufficient to destroy an hypothesis which appears to me to be entirely without foundation, and which, if it were true, would have no other effect than to place Cervantes in M. Lèlut's museum of great men—an honour Cervantes can well do without; for I venture to think he would not be in his right place, although in very good company.

It appears to me, then, that instead of amusing oneself with these useless speculations, it would be infinitely better to follow the example of Doctor Morejon. We have just read his plea, and seen that nothing is wanting to it: the exordium is remarkable, and springs out of the subject. The peroration is eloquent —rather too much so in my opinion—but thus he expresses his own convictions.

That which strikes me most in this curious article, is the number and force of the accumulated proofs in favour of the medical instinct, I might even say the talent of observation of Cervantes. Let me not be mistaken. The author of "Don Quixote" was an observer of vast and profound genius. The half of his life was passed in travelling; consequently he had seen much and he remembered much. Over these elements of all kinds his acute intelligence exercised itself. He spread profusely over these varied recollections all the richness of his imagination, so that to the truth of nature he added the charm and *prestige* of art.

In travelling through the principal universities and the great cities of Europe, Cervantes had certainly not failed to visit, for his instruction, the houses and asylums for the insane, at that time so well regulated in Spain. Lunatics played a considerable part, and held a prominent place in his works. Every one knows that among his moral romances (*novelas ejemplares*), one of the most interesting is that of the licentiate Vidriera (*el licenciado Vidriera*), a poor wretch who had passed his youth in study in the midst of the schools, surrounded by books, resisting all the seductions of love, who, by means of an amorous philter administered by a woman without his knowledge, was thrown into a languishing sickness, and immediately afterwards into a most extraordinary mania. The licentiate took it into his head that his body was made of glass. In consequence of this delusion, he took the most minute and comical precautions to preserve this too fragile exterior from contact with surrounding objects.

These repeated observations of diversified cases of insanity prove not only the sagacity and profoundness of the genius of Cervantes, but they prove also the noble feelings of his excellent soul, which had sympathy for all the misfortunes, and compassion for all the sufferings of man. How well he knew the depths of the human heart, and what a great painter he is in this department! How natural and how true are his pictures! Is there any personage more imaginary, and at the same time more real than "Don Quixote"? He interests and diverts us; he leaves us amused and thoughtful; he makes us dreamers and philosophers; and at the end of his adventures, when the supreme moment arrives, this sublime madman recovers the full use of his reason, he makes himself ready for death with a calm and resigned wisdom which touches us and moves us profoundly. Such is the incomparable art and privilege of genius. Too much time cannot be given to the study of those great authors who have put all their souls into their works, and who represent the age in which they lived. Although the saying of Montesquieu is too witty to be true, it is not less certain that Cervantes may be considered to represent in himself the wit, the manners, the character, and the genius of Spain in the sixteenth century.

As to the work of Dr. Morejon, I refrain from expressing such an opinion upon it as it deserves, leaving it without fear to the judgment of the reader. I will not deny that I think very well of it. I think it new and original, very interesting, extremely curious, and worthy to figure in the best editions of "Don Quixote." It is to be hoped that future editors of this immortal work will give it an honourable position beside the most approved commentaries, such as those of Clémencia and of Navarrete, to which it serves naturally as complement. Whoever shall undertake to write a philosophic history of mental derangement should make a point first of consulting the book of Cervantes and the medico-physiological work of Dr. Morejon.*

---

### Art. IX.—ON THE INSANITY OF CHILDREN.

* The Memoir of Dr. Morejon has been noticed successively by MM. A. de Puebusque and A. de Latour, who have given extracts of it in their valuable works on Spanish literature.

